# Clinical Features of Aortic Dissection in the Emergency Department: A Single-center Experience from South China

**DOI:** 10.5811/westjem.2021.7.52525

**Published:** 2022-06-29

**Authors:** Xiang-Min Li, Guo-Qing Huang, Ai-min Wang, Li-Ping Zhou, Xiao-Ye Mo, Fang-Jie Zhang

**Affiliations:** Xiangya Hospital, Central South University, Department of Emergency Medicine, Changsha, Hunan, People’s Republic of China. National Clinical Research Center for Geriatric Disorders (Xiangya Hospital), Changsha, Hunan, People’s Republic of China

## Abstract

**Objectives:**

Our goal in this study was to determine 1) whether there are any differences in clinical characteristics between Chinese and Western patients with aortic dissection (AD), and 2) the mortality rate of AD patients in the emergency department (ED) and identify the risk predictors for death.

**Methods:**

We retrospectively analyzed patients who were diagnosed with AD and admitted to our ED between September 1, 2017–August 31, 2020. Data on age, gender, clinical manifestation, medical history, routine blood tests, liver and kidney function, coagulation, myocardial enzymology, and mortality were collected.

**Results:**

We enrolled 535 AD patients (422 men and 113 women) with a mean age of 54.7±14.1 years. Type A AD constituted 40% of the total number of AD cases, while type B AD constituted 60%. The proportion of those who were females, 10–92 years, with type A AD, and hypertension in the Chinese population was lower than that in the Western population (P <0.05 for all). Type A AD patients had a higher proportion of acute AD clinical manifestations than did patients with type B AD (P = 0.0084, P <0.05). The mortality rate of type A AD patients (10.75%) was higher than that of type B AD patients (1.87%) (P <0.0001) in the ED. Higher values of white blood cells, neutrophils, high-density lipoprotein, activated partial thromboplastin time, and D-dimer level with worsened hepatic and renal function were found in the deceased group, and multivariate logistic regression revealed that blood urea nitrogen (BUN) levels (P = 0.0031, P <0.05) were significantly associated with death.

**Conclusion:**

In South China, patients with AD had a mean age of 54.7 years, with 78.88% prevalence in males and 66.92% hypertension rate. Type A AD accounted for 40% of all AD cases, and 10.70% of patients with type A AD died in the ED. Elevated BUN levels may be a risk predictor for death in patients with type A AD.

## INTRODUCTION

Until recently, the exact epidemiology of aortic dissection (AD) was not determined, and it was reported that the estimated incidence of AD was six per 100,000 persons per year in the United Kingdom.^1^ The International Registry of Acute Aortic Dissection (IRAD), which was founded in 1996, provides data on large groups of patients with acute AD in the Western population. Several factors, including age, gender, height, weight, and blood pressure, are considered risk factors for AD. The main risk factor for AD is hypertension, which is observed in 65–75% of AD cases, especially in those with poorly controlled blood pressure.^2^ Other risk factors for AD include atherosclerosis (27%); known aortic aneurysm (16%); previous cardiac surgery (16%); Marfan syndrome (5%); iatrogenic causes (4%); and cocaine use (1.8%).^3^ In the IRAD series, 67% of patients presented with type A AD and the remaining 33% with type B. Two-thirds of the patients were men, with a mean age of 63 years.^3^ However, a higher in-hospital mortality was noted among women than among men.^4^

The objectives of the present study were as follows: 1) to determine the clinical characteristics of Chinese patients with type A and B AD and to evaluate whether there were differences with the Western population and 2) to determine possible differences between the mortality of patients with type A and B AD in the emergency department (ED) and to identify high-risk markers for patients with type A AD.

## METHODS

### Study Population

The study protocols were approved by the ethics committee (No. 202012199) of Xiangya Hospital of Central South University and comply with the Declaration of Helsinki. We retrospectively analyzed patients who were diagnosed with AD and admitted to the ED of Xiangya Hospital of Central South University from September 1, 20171–August 31, 2020, regardless of the patient’s initial presentation. The inclusion criteria met one of the following: 1) diagnosis of AD by aortic full-length computed tomography angiography (CTA); 2) diagnosis of AD by magnetic resonance imaging great vessel scan; and 3) a sharp drop in blood pressure when admitted to the ED requiring rescue or cardiac arrest without completing the full-length aortic CTA. Cardiovascular color ultrasound was able to reveal AD in the patients, and the criterion for ultrasound diagnosis in this study was visualization of the dissection flap with or without hemopericardium. Patients with a prior AD surgical history were excluded if CTA did not reveal a new AD. In this study, we used the Stanford AD classification, which divides AD into type A, which involves the ascending aorta, and type B, which does not involve the ascending aorta. According to the 2014 European Society of Cardiology guidelines^5^ on the diagnosis and treatment of aortic diseases, the time course of AD is divided into acute (<14 days), subacute (15–90 days), and chronic (>90 days) phases.

### Data Collection

In this study we used standardized methods for data collection.^5^ First, two trained abstractors who were blinded to the study hypothesis retrospectively collected information regarding the basic identity number and type of patients with diagnosed AD (the imaging reports by the radiologist provided the A and B classification) from the hospital information system during the study period. The two abstractors were trained by the senior author (AMW) on 10 random charts to perform the standardized chart review process, and inconsistent data were reviewed by AMW who independently extracted a sample of 60 visits (11.2%). We assessed the abstractor interrupter reliability for the identity number, and the AD type in this analysis was 0.994. We used a Python-based software (designed by GQH) to capture the data, including patient age, gender, records of lab values, and death in the ED, and two authors (LPZ and XYM) verified whether the captured data was consistent with the hospital information system.

*Population Health Research Capsule*

What do we already know about this issue?

*Aortic dissection (AD) has become common in the ED, with several studies showing the clinical characteristics of AD in South China.*

What was the research question?

*What are the differences in clinical characteristics between Chinese and Western patients with AD, and what is the risk predictor for death?*

What was the major finding of the study?

*In south China, AD patients were younger than the Western population. Elevated blood urea nitrogen may be a risk predictor for death in type A AD.*

How does this improve population health?

*Further research should be conducted on whether there is a pathogenic gene for aortic dissection in Chinese populations.*

Therefore, the basic information of all confirmed AD patients, including age, gender, dissection classification, and whether they were dead or alive in the ED were considered to be accurate and complete. However, not every patient had lab tests in the ED because many patients were already diagnosed in other hospitals and later transferred to our hospital for surgery. Thus, some relevant lab values that were previously reported were not repeated in our department. However, other hospitals’ data was not registered in our hospital information system, which led to missing lab values. Missing data was not included in the analysis.

We collected data for variables including age, gender, clinical manifestation, past medical history, and data on lab values, which included the following: blood routine tests (white blood cells [WBCs]; red blood cells; hemoglobin [HGB]; platelets [PLT]; neutrophil [NC]; and lymphocytes [LC]); liver function (alanine aminotransferase [ALT] and aspartate aminotransferase [AST]); kidney function (serum creatinine [Cr]; blood urea nitrogen [BUN] and uric acid [UA]); coagulation (fibrinogen [FIB]; prothrombin time [PT]; activated partial prothrombin time [APTT]; thrombin time [TT]; international normalized ratio [INR]; fibrinogen degradation products [FDP] and D-dimer); myocardial enzymology (creatine kinase [CK]; creatine kinase isoenzyme [CK-Mb]; lactate dehydrogenase [LDH] and myoglobin [Mb]); total bilirubin (TBIL); triglycerides (TG); total cholesterol (TC); high-density lipoprotein (HDL); low-density lipoprotein (LDH); and C-reactive protein (CRP). All lab work was performed within the first hour after the patient was admitted to the ED. We also collected the mortality and survival data of patients diagnosed with AD in the ED. Data was collected from 1) patients who died in the ED, 2) patients who did not die in the ED and were transferred to the cardiovascular or vascular surgery ward, and 3) patients who did not die in the ED but were discharged upon the patient’s or their guardian’s request. For this study we did not collect data on whether the patient survived after surgery or discharge.

### Statistical Analysis

We performed all statistical analyses using GraphPad Prism 9 (GraphPad Software, Inc, San Diego, CA). The results are expressed as mean ± standard deviation (SD). Continuous variables were compared using Student’s t-test for normal distributions and the Mann-Whitney U test for non-normal distributions. Comparisons of rates between groups were performed using the chi-square test or Fisher’s exact test. We performed multivariate logistic regression analyses to identify the independent factors related to the deceased group. The threshold for the entry of variables into the multivariate models was *P* <0.0001. We also calculated odds ratios (OR) and 95% confidence intervals (CI). Statistical significance was set at *P* <0.05.

## RESULTS

We included a total of 535 (422 [78.88%] male, 113 [21.12%] female) AD patients in the study, with a mean age of 54.7±14.1 years (range: 10–92 years), as shown in Table 1. The incidence of type A AD was 40%, while that of type B AD was 60%. We compared our data with those reported in previous studies in the IRAD.^7^ The AD patients in our study were younger than the Western population (*P* <0.0001). Furthermore, our study indicated that male patients had higher values of total AD than those in the Western population (*P* <0.0001). The classification of AD in our study population is significantly different from that in a study on a Western population by Pape et al.^7^ (*P* <0.0001)

Our study showed that hypertension was the most common risk factor associated with AD, with 137 (64.02%) Type A AD patients with hypertension and 221 (68.85%) Type B AD patients with hypertension (Table 2). The difference between the rates of hypertension in type A and type B AD was not significant (*P* >0.05). Patient history is shown in Table 3.

Table 4 lists the clinical symptoms of patients with AD. More than 30 different symptoms at onset and during progression were observed. Patients with type A AD had a higher rate of chest pain than those with type B AD (*P* <0.0001), while abdominal pain was more common in patients with type B AD than in patients with type A AD (*P* <0.0001). Other chest symptoms, including chest tightness and shortness of breath, were more common in patients with type A AD than in those with type B AD (*P* <0.0001, *P* <0.05, respectively). Central nervous system symptoms were higher in the type A AD group than in the type B AD group (*P* <0.05). Furthermore, the onset of acute myocardial infarction appeared to be an initial presentation that was higher in type A than in type B AD (*P* <0.05).

Among a total of 535 patients, 511 had a recorded duration of symptom onset when admitted to our ED. Of the 201 patients with a recorded duration among 214 patients with type A AD, 194 were from the acute AD group, four were from the subacute AD group, and three were from the chronic AD group. There were 310 patients with a recorded duration time among 321 type B AD patients, with 280 from the acute AD group, 22 from the subacute AD group, and eight from the chronic AD group. The chi-square test indicated that patients with type A AD had a higher proportion of acute AD clinical manifestations than those with type B AD (*P* <0.05).

Subsequently, we analyzed the mortality rate of 214 patients with type A AD in the ED. Sixteen male (aged 53.6±12.7 years) and seven female (aged 57.6±15.7 years) patients died in the ED. There were no age- or gender-related differences in the mortality rates of patients with type A AD (*P* >0.05, respectively). Of the 321 type B AD patients, there were five males and one female who died in the ED. The mortality rate in type A AD patients was higher than that in type B AD patients in the ED (*P* <0.0001). Among the 23 patients with type A AD who died, 18 died within 24 hours (h) of symptom onset, two died within 48 h of symptom onset, one died within 72 h of symptom onset, and one died 10 days from symptom onset. One patient did not have a record of the duration from symptom onset to admission in the ED, but the patient died 4 h after admission to the ED.

Due to the higher mortality rate of Type A AD, we analyzed the data of the deceased and survivor patients (Table 5). Of the 191 patients in the surviving group 161 had complete data, while 15 of the 23 patients in the deceased group had complete data. The 23 patients in the deceased group, with a mean age of 60.1±12.8 years, were older than the 191 survivors, who had a mean age of 54.0±13.7 years (*P* <0.05). The values of WBC, NC, HDL, APTT, and D-dimer were all higher in the deceased group than in the surviving group, and the HGB levels were lower in the deceased group than in the surviving group. As a reflection of organ function, BUN, Cr, UA, ALT, AST, CK, CK-MB, LDH, and Mb were all higher in the deceased group than in the surviving group.

We performed multivariate logistic regression analyses to determine independent factors related to the deceased patient group. The threshold for the entry of variables into multivariate models was *P* <0.0001. The results of the multivariate logistic regression analysis are shown in Table 6. We found that BUN levels (*P*<0.01; OR, 0.8408) were significantly associated with death.

## DISCUSSION

Aortic dissection is a life-threatening vascular disease associated with high morbidity, and most patients with AD are diagnosed in the ED. In our study, 29 (5.42% of all patients with AD) died in the ED. There was a relatively low mortality rate of 10.70% among patients with type A AD who died in the ED. Among the 23 deceased type A AD patients, 18 (78%) died within the first 24 h, while the others died during the acute course. In Japan, the prehospital mortality rate is 61.4%. Combining prehospital with in-hospital mortality rates shows that 93% of deaths from AD occur within 24 h after onset.^8^ A previous study showed that 22.7% of the hospitalized patients died within the first 6 h, 33.3% within 12 h, 50% within 24 h, and 68.2% within the first two days after admission.^8^ The reason for the relatively low mortality in our ED may have been because many dissection patients died at home or on the way to the hospital. Moreover, in our study, the average age of patients with AD was lower than that among Western patients. Japanese AD patients exhibited a peak in AD at 70 years of age,^8^ with younger patients having relatively healthier bodies and fewer comorbidities.

Emergency physicians are familiar with the diagnosis and treatment of AD, and patients with chest and abdominal symptoms as the first manifestation rarely underwent only radiographic examinations; they almost always had CT performed. Especially for patients with unexplained chest and abdominal pain, AD could be diagnosed quickly. In the ED, AD patients received active blood pressure and heart rate control,^10^,^11^ and cardiovascular and vascular surgeries were quickly informed, which reduced the time to surgery and hospital admission, all of which led to decreased mortality in AD patients. Deceased patients with type A AD had the following characteristics: older age; higher WBC, NC, HDL, APTT, and D-dimer levels; lower HGB; and remarkable organ dysfunction, including impairments to renal function (BUN and Cr), liver function (ALT and AST), and myocardium (CK, CK-MB, LDH, and Mb), while the BUN level appeared to be a predictor of death.

A previous study in China demonstrated that the WBC count is a potential independent risk factor for in-hospital death in type A AD patients.^12^ Another study also indicated that increased Cr, BUN, and AST levels were significantly related to a higher rate of in-hospital mortality in patients with type A AD.^13^ The WBC and NC elevation might be a result of the effects of a systemic inflammatory response syndrome in AD pathology.^8^ Type A AD could affect the ascending aorta and even extend to the full length of the aorta, leading to organ dysfunction in any involved vascular area of the body, which might be the reason for organ dysfunction.^7^

With increased economic development and improvements in living standards, both hypertension and atherosclerosis are becoming more common in the Chinese population. This has led to an increase in the number of AD patients, and with improvements in physicians’ awareness of AD and the high availability of CT, the diagnosis rate of AD has also been increasing.^14^ However, our results indicated that AD in South China showed differences with AD in Western populations. In South China, the number of type B AD patients was higher than that of type A AD patients, while in Western populations the number of type B AD patients was lower than that of type A AD patients. Furthermore, in our study, Chinese AD patients presented their initial symptoms at an average age of 54 years, which is lower than that of Western populations by roughly 10 years and lower than that of Japanese AD patients by approximately 15 years.^8^

Our results were in line with those of another Chinese study^14^ that included 1003 AD patients with a mean age of 52 years and that had a higher number of type B AD patients than type A AD patients. The previous study,^14^ the first Registry of Aortic Dissection in China (Sino-RAD), showed that the Chinese population was different from European and American populations in terms of age of onset, gender, clinical manifestations, and fatality rate. However, there have been no new AD data reports in the past seven years, although Sino-RAD has described a number of pathogenic characteristics of the Chinese people.^15^ Nevertheless, the reason for the difference between the Chinese and Western populations is still unclear. We speculate that this difference might be associated with China’s large geographic area and uneven economic development. Many hypertensive patients are not detected at an early stage and are actively treated and well controlled,^16^ while uncontrolled hypertension remains the most significant treatable risk factor for acute AD.^1^ Additionally, racial differences should also be considered; for example, Black patients were younger with a higher prevalence of type B AD (52.4%) than those in White patients.^17^ Furthermore, in China every county hospital was equipped with CT, and almost all patients underwent CT examination when the patient had an unexplained chest pain or abdominal pain. These CT tests might have increased the detection rate of AD.

Our study showed that hypertension was most common in patients with AD. However, the results indicated that the hypertension rate in patients with AD in South China was lower than that in Western populations, with a hypertension rate of 76.6% as the most common risk factor. Smoking, drinking, and coronary atherosclerotic heart disease were also common in both type A and type B AD patients. These results are in line with a previous Chinese investigation that showed that smoking and drinking were the common risk factors for Chinese AD patients.^14^ However, other risk factors such as Marfan syndrome, connective tissue disease, diabetes, and history of AD surgery for heart surgery were not common in the South China AD patients, while previous cardiac surgery, Marfan syndrome, and cocaine use have been implicated in Western patients.^3^ The reasons for the differences in these risk factors between the Chinese and Western populations may be related to differences in eating habits and body mass index, which may have led to a lower incidence of hypertension in the Chinese population than in the Western population.^18^,^19^ Moreover, racial differences and the relatively small sample sizes should also be considered.

In our study we discerned more than 30 different symptoms at onset and during progression; therefore, it was not easy to diagnose AD immediately based only on the symptoms. The most common symptom was pain: type A AD patients had a higher degree of chest pain and other chest symptoms, including chest tightness and shortness of breath, while abdominal pain was more common in patients with type B AD. Central nervous system symptoms and onset of acute myocardial infarction were also more common in type B AD. The differences in symptoms between type A and type B AD are related to the location of the dissection tear. Because type A AD can involve the full length of the aorta, it leads to more diverse clinical manifestations in patients with type A AD.^5^,^7^,^20^

## LIMITATIONS

First, we retrospectively analyzed the data of only our ED patients; date of patients admitted through outpatient procedures were not collected because patients in our hospital came from the Hunan province and neighboring 2–3 provinces. Thus, the epidemiology of AD could not be accurately estimated. Second, we had no follow-up data on signed and discharged patients and hospitalized patients, and the mortality rate of AD might, therefore, be lower than the actual rate. Third, this study did not explore the effects of drugs on mortality and laboratory test results. Some patients received drug treatment before admission. For example, patients with previous coronary atherosclerotic heart disease may have had oral aspirin, clopidogrel, or ticagrelor, and patients who had undergone heart surgery in the past may have been taking oral warfarin, and these drugs might have affected the lab values. Our results indicated that the ages at which the incidence of AD and the hypertension rate peaked were lower than those in Western countries; however, the possible reasons for this observation were not determined, and further research should focus on exploring whether the differences between the genetic characteristics and living habits of Chinese and Western people cause differences in AD characteristics.

## CONCLUSION

In South China, AD patients had a mean age of 54.7 years, 78.88% of whom were male and had a hypertension rate of 66.92%. Type A AD patients constituted 40% of all AD patients, and 10.70% of type A AD patients died in the ED. Elevated blood urea nitrogen levels might be a risk predictor for death in patients with type A AD.

## Figures and Tables

**Table 1:** Gender, classification and age differences between South China and Western populations (IRAD^*^ data).

	South China data (N = 535)	IRAD data^7^ (N = 4,428)	*P* value
Gender			
Male	422	2,964	<0.0001
Female	113	1,464	
Classification			
Type A AD	214	2,952	<0.0001
Type B AD	321	1,476	
Age			
Total AD	54.7±14.1	62.2 ±14.5	All *P* <0.0001
Type A AD	54.7±13.7 (n = 214)	61.5±14.6 (n = 2,952)^7^	
Type B AD	58.0±14.2 (n = 321)	63.6±14.1 (n = 1,476)^7^	

*IRAD*, International Registry of Aortic Dissection; *AD*, aortic dissection.

**Table 2:** Differences in total hypertension rate in aortic dissection patients.

	South China population	Western population^7^	Total	P value
Hypertension in AD	358	3,247	3,605	<0.01
Non-hypertension in AD	177	1,181	1,358	
Hypertension in Type A AD	137	2,089	2,226	<0.0001
Non-hypertension in Type A AD	77	2,952	3,029	
Hypertension in Type B AD	221	1,158	1,379	<0.001
Non-hypertension in Type B AD	100	318	418	

*AD*, aortic dissection.

**Table 3:** Patients’ istories.

	Type A AD	Type B AD	P value
Patients	214	321	
Age	54.7±13.7	58.0±14.2	<0.01
Gender (male,%)	157,73.36%	265,82.55%	<0.01
Connective tissue disease	1	1	1.00
Drinking	12	18	1.00
History of AD surgery	6	9	1.00
Familial AD	1	2	1.00
Pregnancy	2	2	1.00
Previous cerebral infarction	5	6	0.76
COPD	2	5	0.71
Smoking	47	65	0.67
History of AD without surgery	1	4	0.65
Atrial fibrillation	2	1	0.57
Bradycardia	2	1	0.57
CKD5	6	13	0.49
Scoliosis	1	0	0.40
Marfan syndrome	3	1	0.31
Diabetes	8	7	0.30
Hypertension (%)	137 (64.02%)	221 (68.85%)	0.26
Coronary atherosclerotic heart disease	18	39	0.20
History of heart surgery	4	1	0.09
Died in ED	23, 10.75%	6, 1.87%	<0.0001

*AD*, aortic dissection; COPD, chronic obstructive pulmonary disease; *CKD*, chronic kidney disease; *ED*, emergency department.

**Table 4:** Signs and symptoms in aortic dissection patients.

Clinical symptoms	TAAD (N = 214)	TBAD (N = 321)	P value
Chest pain	109	93	<0.0001
Back pain	14	26	0.62
Chest and back pain	30	53	0.47
Abdominal pain	19	86	<0.0001
Chest tightness	25	7	<0.0001
Dizziness and headache	6	8	1.00
Cough	2	2	1.00
Hoarseness	0	1	1.00
Unclear speech	0	1	1.00
Shock	0	1	1.00
Hemoptysis	1	3	0.65
Hematemesis	0	2	0.52
Hematuria	0	2	0.52
Obstipation	0	2	0.52
Painful urination	0	2	0.52
Cardiac arrest	1	0	0.40
Twitch	1	0	0.40
Asymptomatic	3	10	0.26
Vomiting	4	2	0.22
Limb weakness or pain	10	8	0.22
Abdominal fullness	0	5	0.16
Coma	2	0	0.16
Neck pain	2	0	0.16
Fever	2	0	0.16
Bloody stools	1	7	0.15
Syncope	7	3	0.10
Palpitations	4	1	0.09
Onset as new cerebral infarction	4	1	0.09
Waist pain	0	6	0.09
Weariness	0	6	0.09
Post-trauma	0	6	0.09
Shortness of breath	10	3	<0.01
Confusion	12	4	<0.01
Onset as acute myocardial infarction	6	1	0.02

*TAAD*, type A aortic dissection; *TBAD*, type B aortic dissection.

**Table 5:** The differences between surviving and deceased patients in Type A AD.

	Survivor	Deceased	P value
Age	54.0±13.7	60.1±12.8	0.04
WBC	11.78±0.37	14.35±1.23	0.04
HGB	126.2±1.78	111.9±8.7	0.02
PLT	181.1±6.30	175.9±20.67	0.08
NC	9.71±0.36	12.51±1.14	0.02
LC	1.13±0.53	0.94±0.09	0.28
BS	7.61±0.16	8.77±1.50	0.11
BUN	7.52±0.34	13.23±1.45	<0.0001
Cr	124.1±9.97	247.9±42.95	<0.001
UA	384.5±11.74	540.9±55.31	<0.001
TBIL	17.80±0.93	20.90±3.57	0.35
TC	4.36±0.92	4.25±0.25	0.71
TG	1.79±0.16	1.65±0.20	0.79
HDL	1.09±0.28	1.77±0.70	0.01
LDL	2.69±0.56	2.48±0.23	0.30
CK	214.1±40.26	549.1±278.4	0.03
LDH	289±29.77	2278±1517	<0.0001
CKMB	19.35±1.98	112.6±66.05	<0.0001
Mb	155.8±40.39	905.4±442.5	<0.0001
ALT	64.29±17.76	643.5±482	<0.01
AST	85.65±27.03	743.5±439.2	<0.0001
CRP	37.63±4.44	28.11±14.94	0.61
PT	15.65±0.71	18.92±2.59	0.21
APTT	34.77±1.13	49.85±11.26	<0.01
TT	202.7±1.46	23.66±3.72	0.52
FIB	3.37±0.17	2.94±0.49	0.47
INR	1.26±0.64	1.53±.024	0.23
FDP	27.53±2.41	43.56±10.09	0.07
D-dimer	2.17±0.17	4.16±0.87	<0.01

*WBC*, white blood count; *HGB*, hemoglobin; *PLT*, platelet count; *NC*, neutrophil count; *LC*, lymphocyte count; *BS*, blood sugar; *BUN*, blood urea nitrogen; *Cr*, creatine levels; *UA*, urinalysis; *TBIL*, total bilirubin; *TC*, total cholesterol; *TG*, triglycerides; *HDL*, high-density lipoprotein; *LDL*, low-density lipoprotein; *CK*, creatine kinase; *LDH*, lactate dehydrogenase; *CKMB*, creatine kinase-MB; *Mb*, myoglobin; *ALT*, alanine aminotransferase; *AST*, aspartate aminotransferase; *CRP*, C-reactive protein; *PT*, prothrombin time; *APTT*, activated partial thromboplastin time; *TT*, thrombin time*; FIB*, fibrinogen; *INR*, international normalized ratio; *FDP*, fibrin degradation products.

**Table 6:** Multivariate logistic regression models for independent factors related to patient deaths.

	P	OR	95% CI
BUN	<0.01	0.8408	0.7459 – 0.9441
LDH	0.25	0.9988	0.9949 – 1.000
CKMB	0.07	0.9841	0.9644 – 1.001
Mb	0.23	1.001	0.9997 – 1.002
AST	0.33	1.002	0.9997 – 1.008

*OR*, odds ratio; *CI*, confidence interval; *BUN*, blood urea nitrogen; *LDH*, lactate dehydrogenase; *CKMB*, creatine kinase-MB; *AST*, aspartate aminotransferase.
